# AMPK Signalling in Heart Failure: From Metabolic Sensor to Context-Dependent Therapeutic Target

**DOI:** 10.3390/biomedicines14061362

**Published:** 2026-06-17

**Authors:** Rayan Arzouni, Reem Aazar, Seif Asakrieh, Seif Cattan, Aleksandar Jovanović

**Affiliations:** 1 Department of Basic and Clinical Sciences, University of Nicosia Medical School, Nicosia CY-1700, Cyprus; arzouni.r@live.unic.ac.cy (R.A.); aazar.r@live.unic.ac.cy (R.A.); cattan.s@live.unic.ac.cy (S.C.); 2 Faculty of Medicine, University of Debrecen, 4032 Debrecen, Hungary; seif-asakrieh@mailbox.unideb.hu; 3 Center for Neuroscience and Integrative Brain Research (CENIBRE), University of Nicosia, Nicosia CY-1700, Cyprus

**Keywords:** AMPK, heart failure, metabolism, mitochondrial dysfunction, autophagy, HFpEF, HFrEF, SGLT2 inhibitors, metformin, precision medicine

## Abstract

Heart failure (HF) is a complex clinical syndrome characterized not only by impaired cardiac function but also by profound disturbances in myocardial energy metabolism. AMP-activated protein kinase (AMPK), a central cellular energy sensor, plays a critical role in maintaining metabolic homeostasis by coordinating pathways involved in substrate utilization, mitochondrial function, autophagy, and stress adaptation. Experimental evidence supports a cardioprotective role of AMPK activation, including improved energetic efficiency, attenuation of pathological remodeling, and enhanced cellular resilience. However, emerging data indicate that AMPK signaling is highly context-dependent, with its effects varying according to HF phenotype, disease stage, and isoform-specific activity. While indirect AMPK modulation through established therapies such as metformin and sodium-glucose cotransporter 2 (SGLT2) inhibitors has demonstrated clinical benefit, the specific contribution of AMPK to these effects remains incompletely defined. Furthermore, direct pharmacological activation is limited by challenges including tissue specificity, off-target effects, and potential adverse outcomes associated with sustained activation. This review provides a comprehensive overview of AMPK signaling in HF, focusing on its role in metabolic remodeling, mitochondrial regulation, and interaction with key cardioprotective pathways. We also examine current clinical and translational evidence and discuss emerging strategies aimed at achieving isoform-selective and tissue-specific modulation. Collectively, these insights support a shift from broad AMPK activation toward precision-based therapeutic approaches tailored to the disease context.

## 1. Introduction

Heart failure (HF) remains a leading cause of morbidity and mortality worldwide, affecting tens of millions of individuals and placing a substantial burden on healthcare systems [1,2]. Traditionally defined as a syndrome of impaired ventricular filling and/or ejection, HF is increasingly recognized as a disorder that extends beyond hemodynamic dysfunction to include profound alterations in myocardial metabolism and cellular homeostasis [2,3]. Current clinical classification stratifies HF according to left ventricular ejection fraction into heart failure with reduced ejection fraction (HFrEF), mildly reduced ejection fraction (HFmrEF), and preserved ejection fraction (HFpEF) [1]. While clinically useful, this framework reflects substantial biological heterogeneity, particularly in metabolic remodeling and systemic comorbidities [4]. HFpEF, for example, is frequently associated with obesity, insulin resistance, and systemic inflammation [5,6], whereas HFrEF is more commonly linked to ischemic injury and contractile dysfunction [4]. These distinctions have important implications for therapeutic responsiveness.

Recent advances in cardiovascular metabolism have established energetic dysfunction as a central driver of HF progression rather than merely a secondary consequence of cardiac dysfunction. Experimental, translational, and clinical investigations have demonstrated that disturbances in substrate utilization, mitochondrial quality control, redox balance, and intracellular signaling pathways directly contribute to adverse cardiac remodeling and disease progression [3,7]. Within this evolving paradigm, AMP-activated protein kinase (AMPK) has emerged as one of the most extensively studied metabolic regulators in cardiovascular biology [8,9]. Over the past two decades, evidence from genetic models, pharmacological studies, and disease-specific investigations has implicated AMPK in the regulation of myocardial energetics, mitochondrial function, autophagy, inflammation, fibrosis, and vascular homeostasis [8,9,10,11,12,13,14,15]. Furthermore, growing interest in therapies with known AMPK-activating properties, including metformin and sodium-glucose cotransporter-2 inhibitors, has intensified efforts to define the contribution of AMPK signaling to improved HF outcomes [7,8].

The myocardium is among the most energy-demanding tissues in the body, relying predominantly on mitochondrial oxidative phosphorylation to sustain continuous ATP production [15]. Under physiological conditions, the heart exhibits remarkable metabolic flexibility, utilizing fatty acids, glucose, lactate, and ketone bodies depending on substrate availability [3]. During HF progression, this flexibility is progressively impaired [2]. Early adaptive responses often involve a shift from fatty acid oxidation toward increased glucose utilization; however, advanced HF is characterized by reduced oxidation of both substrates, mitochondrial dysfunction, and overall energy deficiency [2,3].

AMP-activated protein kinase (AMPK) is a highly conserved serine/threonine kinase that functions as a central regulator of cellular energy balance [10,11,12]. Activated in response to increases in the AMP/ATP and ADP/ATP ratios, AMPK coordinates metabolic pathways to restore energy homeostasis by promoting ATP-generating processes while inhibiting energy-consuming anabolic pathways [10,11]. Beyond metabolism, AMPK regulates mitochondrial biogenesis, autophagy, oxidative stress responses, and cellular survival pathways [13,14,15]. Given these roles, AMPK has emerged as a key mediator of cardiac adaptation to energetic stress and a potential therapeutic target in HF [8]. However, its biological effects are complex and context-dependent [8]. While acute activation generally supports adaptive responses, sustained or non-selective activation may lead to unintended consequences, including excessive catabolism or impaired cellular growth [8]. Moreover, AMPK exists as multiple isoforms with tissue-specific functions, further complicating its therapeutic targeting [16].

Despite the extensive literature supporting AMPK-mediated cardioprotection, several important questions remain unresolved. Current evidence suggests that AMPK signaling may exert distinct effects across different HF phenotypes, disease stages, and cellular compartments, yet the molecular mechanisms underlying these context-dependent responses remain incompletely understood [3,7,16]. Emerging studies further indicate isoform-specific functions of AMPK subunits in cardiomyocytes, fibroblasts, endothelial cells, and immune cells, highlighting a level of biological complexity not fully addressed in earlier reviews [16]. In addition, significant translational challenges remain, including uncertainty regarding the optimal timing, duration, tissue specificity, and magnitude of AMPK activation required for therapeutic benefit in HF [8,16].

Several reviews have comprehensively summarized the fundamental metabolic functions of AMPK and its established cardioprotective actions [8,9,13,14,15]. However, major advances over the last several years—including improved understanding of HF phenotypic heterogeneity, cardiac intermediary metabolism, isoform-specific AMPK signaling, mitochondrial quality-control pathways, and interactions between AMPK and contemporary HF therapies—have substantially expanded the field [3,7,16]. Therefore, the objective of the present review is not only to summarize established mechanisms of AMPK-mediated cardioprotection but also to critically evaluate recent advances, identify persistent knowledge gaps, and discuss future opportunities and challenges associated with therapeutic targeting of AMPK in HF. This focus distinguishes the present review from previous publications and provides an updated perspective on the translational potential of AMPK-based interventions in modern HF management.

## 2. Structure and Activation of AMPK

**Figure 1:**
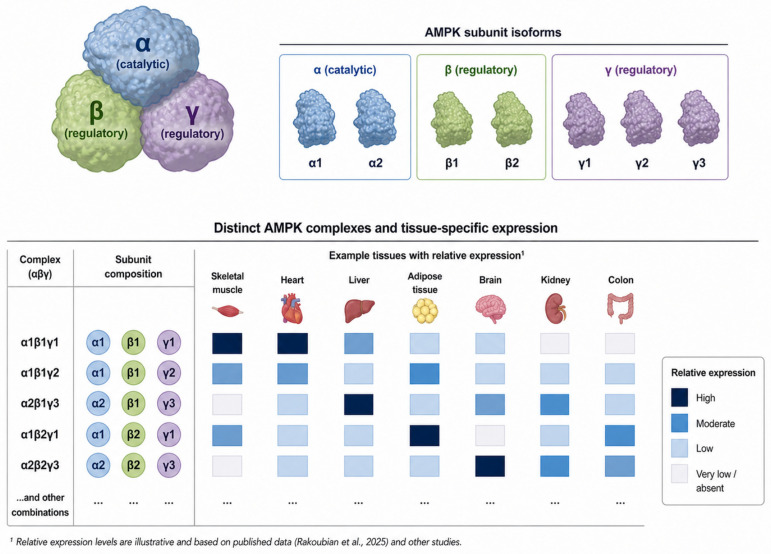
AMPK heterotrimeric structure and isoform diversity. AMPK is a heterotrimeric serine/threonine kinase composed of one catalytic α-subunit and two regulatory subunits, β and γ. Each subunit exists as multiple isoforms (α1/α2, β1/β2, γ1/γ2/γ3), which combine to form distinct AMPK complexes with tissue-specific expression patterns and functional specialization. The schematic illustrates the structural organization of the AMPK complex, representative isoform combinations, and relative expression across metabolically active tissues including skeletal muscle, heart, liver, adipose tissue, brain, kidney, and colon. Differences in isoform composition contribute to context-dependent regulation of cellular metabolism and stress adaptation in health and disease.

AMPK is a heterotrimeric complex composed of a catalytic α-subunit and two regulatory subunits, β and γ [10,11,12]. Each subunit exists in multiple isoforms (α1/α2, β1/β2, γ1/γ2/γ3), which combine to form distinct complexes with tissue-specific expression patterns [17] (Figure 1). In cardiac and skeletal muscle, the α2 isoform is predominant, suggesting a particularly important role in tissues with high energetic demand [16]. The α-subunit contains the kinase domain responsible for catalytic activity, including a critical activation loop in which phosphorylation at threonine 172 (Thr172) is essential for full activation [10,12]. It also includes an autoinhibitory domain that suppresses kinase activity under basal conditions, and a C-terminal domain that mediates interactions with the β- and γ-subunits [10]. The β-subunit serves as a scaffold linking the α- and γ-subunits and contains a carbohydrate-binding module that enables interaction with glycogen particles, potentially linking AMPK activity to cellular energy storage [11]. The γ-subunit contains four cystathionine β-synthase domains that bind adenine nucleotides (AMP, ADP, and ATP), allowing AMPK to sense changes in cellular energy status [7,17] (Figure 1).

Under physiological conditions, AMPK activity is relatively low due to autoinhibitory interactions within the α-subunit [10]. Activation occurs in response to energetic stress, reflected by increased intracellular AMP and ADP levels relative to ATP [12]. Binding of AMP or ADP to the γ-subunit induces conformational changes that promote phosphorylation of Thr172 and protect the enzyme from dephosphorylation, thereby sustaining its active state [12]. While AMP has traditionally been considered the primary activator, evidence suggests that ADP also plays an important role in stabilizing AMPK activation under physiological conditions [12].

Phosphorylation of AMPK is mediated primarily by upstream kinases, most notably liver kinase B1 (LKB1), which responds to energy depletion, and Ca^2+^/calmodulin-dependent protein kinase kinase β (CaMKKβ), which is activated in response to increases in intracellular calcium [18,19]. These pathways are not mutually exclusive and may act in parallel depending on cellular context, linking AMPK activation to both metabolic and calcium-dependent signaling [19].

Beyond its canonical upstream kinases, AMPK functions within a highly interconnected signaling network that integrates metabolic stress, redox homeostasis, inflammation, and cellular defense responses. In addition to LKB1 and CaMKKβ, transforming growth factor-β-activated kinase 1 (TAK1) has been reported to activate AMPK under specific stress and inflammatory conditions, providing a mechanistic link between immune signaling and cellular energy sensing [20]. These observations suggest that AMPK operates not only as a metabolic sensor but also as a broader coordinator of cellular stress adaptation.

A particularly important downstream interaction involves the nuclear factor erythroid 2–related factor 2 (NRF2) pathway, a master regulator of antioxidant defence. Increasing evidence indicates bidirectional crosstalk between AMPK and NRF2 signaling. AMPK activation can promote NRF2 nuclear translocation and transcriptional activity either directly or indirectly through inhibition of glycogen synthase kinase-3β (GSK3β), thereby enhancing the expression of antioxidant enzymes such as heme oxygenase-1 (HO-1), superoxide dismutase, catalase, and NAD(P)H quinone oxidoreductase 1 [21,22]. Conversely, NRF2-mediated preservation of redox homeostasis may support mitochondrial function and sustain AMPK signaling under conditions of oxidative stress. Through this AMPK–NRF2 axis, cells can coordinate energy homeostasis with antioxidant defence mechanisms.

AMPK also exhibits extensive interactions with inflammation-associated signaling pathways. Activation of AMPK generally suppresses pro-inflammatory responses through inhibition of nuclear factor-κB (NF-κB) signaling and attenuation of NLRP3 inflammasome activation [23,24]. These effects reduce the production of inflammatory mediators including tumor necrosis factor-α, interleukin-1β, and interleukin-6, which are recognized contributors to adverse cardiac remodeling and HF progression. In parallel, AMPK activation promotes autophagy and mitochondrial quality control, thereby limiting the accumulation of damaged mitochondria that can serve as important sources of inflammatory signaling.

The relationship between AMPK and oxidative stress is similarly complex. Reactive oxygen species (ROS) can activate AMPK through both direct and indirect mechanisms, including alterations in AMP/ATP ratios and redox-sensitive upstream kinases [25]. Once activated, AMPK exerts antioxidant effects by enhancing mitochondrial biogenesis through the PGC-1α pathway, stimulating autophagic clearance of dysfunctional mitochondria, and promoting NRF2-dependent antioxidant gene expression [11,21,22]. Consequently, AMPK is increasingly recognized as a central node linking mitochondrial function, oxidative stress responses, inflammation, and cellular survival.

Another major downstream target of AMPK is the mechanistic target of rapamycin (mTOR) pathway. Through phosphorylation of TSC2 and RAPTOR, AMPK inhibits mTOR complex 1 activity, thereby suppressing anabolic processes and promoting autophagy during energetic stress [26]. The balance between AMPK and mTOR signaling is increasingly viewed as a critical determinant of cardiac adaptation or maladaptation during HF progression, obesity, and diabetes.

In addition to physiological activation, AMPK can be modulated pharmacologically. Indirect activators, such as metformin, increase the AMP/ATP ratio by modulating mitochondrial function, whereas direct activators bind to allosteric sites on the enzyme complex [27,28,29]. However, many pharmacological agents lack specificity for particular AMPK isoforms or tissues, and their long-term effects remain incompletely understood [29].

Importantly, emerging evidence suggests that not only the magnitude of AMPK activation but also its spatial, temporal, and isoform-specific characteristics determine its functional outcomes [11,19]. This complexity underlies many of the challenges associated with targeting AMPK therapeutically in heart failure.

## 3. AMPK-Mediated Metabolic and Cellular Mechanisms

### 3.1. Metabolic Reprogramming

AMPK is a central regulator of myocardial metabolism, coordinating pathways that balance energy production and consumption [10,12]. Upon activation, AMPK enhances glucose uptake by translocating glucose transporters, such as GLUT1 and GLUT4, to the plasma membrane and stimulates glycolysis by activating phosphofructokinase-2, thereby increasing ATP production under conditions of energetic stress [10,12]. Simultaneously, AMPK suppresses energy-consuming anabolic processes, including glycogen synthesis and lipid biosynthesis, thereby conserving ATP [10]. While these effects are beneficial in acute stress conditions, prolonged suppression of anabolic pathways may impair cellular repair and structural maintenance [2].

AMPK also promotes fatty acid oxidation through inhibition of acetyl-CoA carboxylase, resulting in reduced malonyl-CoA levels and increased mitochondrial uptake of fatty acids [10,12]. In parallel, lipid synthesis is inhibited through suppression of sterol regulatory element-binding proteins [10]. Although these mechanisms enhance energy efficiency, their long-term impact in HF remains complex, particularly in advanced disease stages characterized by metabolic inflexibility [2,3].

### 3.2. Mitochondrial Regulation

Mitochondrial dysfunction is a hallmark of HF and contributes to impaired ATP production and increased oxidative stress [3]. AMPK plays a key role in maintaining mitochondrial homeostasis by promoting mitochondrial biogenesis, improving oxidative metabolism, and regulating reactive oxygen species (ROS) production [13,15]. Activation of AMPK stimulates peroxisome proliferator-activated receptor gamma coactivator-1α (PGC-1α), thereby enhancing mitochondrial number and function [15]. In addition, AMPK improves the efficiency of the electron transport chain, reducing electron leakage and limiting ROS generation [13]. While these effects are generally protective, ROS also serve important signaling roles, and excessive suppression may interfere with physiological cellular processes [13]. Therefore, AMPK-mediated modulation of oxidative stress must be considered within a balanced regulatory framework.

### 3.3. Autophagy and Mitophagy

AMPK is a key regulator of autophagy, a cellular process responsible for the degradation and recycling of damaged proteins and organelles [14]. It promotes autophagy through inhibition of mechanistic target of rapamycin complex 1 (mTORC1) and direct activation of Unc-51-like kinase 1 (ULK1) [14,30].

Mitophagy, a specialized form of autophagy targeting damaged mitochondria, is also regulated by AMPK through pathways involving PINK1 and Parkin [31]. This process is essential for maintaining mitochondrial quality and preventing the accumulation of dysfunctional organelles.

However, autophagy exhibits a dual role in cardiac physiology. While moderate activation supports cellular homeostasis, excessive or dysregulated autophagy may contribute to cardiomyocyte loss and disease progression [30]. Consequently, the effects of AMPK on autophagy are highly dependent on disease stage and cellular context.

### 3.4. Anti-Hypertrophic and Anti-Fibrotic Effects

Cardiac hypertrophy initially represents an adaptive response to increased workload but becomes maladaptive when sustained, contributing to ventricular remodeling and functional decline. AMPK has been shown to attenuate hypertrophic signalling by inhibiting mTORC1 and modulating protein synthesis pathways [32]. In experimental models, AMPK activation reduces cardiomyocyte size and limits pathological remodeling, effects that are partly mediated through enhanced autophagy and improved energy balance [32].

In addition to its anti-hypertrophic role, AMPK influences cardiac fibrosis, a key determinant of myocardial stiffness and impaired relaxation. Activation of AMPK has been associated with reduced fibroblast proliferation and decreased extracellular matrix deposition, in part through inhibition of transforming growth factor-β (TGF-β) signaling pathways [33,34]. Regulation of antifibrotic microRNAs, including the miR-29 family, has also been implicated [33].

Despite these promising findings, most evidence is derived from preclinical studies, and the extent to which AMPK-mediated anti-fibrotic effects translate into clinically meaningful outcomes remains uncertain.

### 3.5. Anti-Inflammatory Effects

Inflammation plays a central role in HF progression, contributing to myocardial injury, fibrosis, and contractile dysfunction. AMPK modulates inflammatory signaling through several mechanisms, most notably by inhibiting nuclear factor-κB (NF-κB) [35].

Through this pathway, AMPK reduces the production of cytokines such as interleukin-6 and tumor necrosis factor-α [35]. In addition, AMPK interacts with regulators including SIRT1 and PGC-1α, linking metabolic status to inflammatory responses [35]. AMPK also suppresses activation of the NLRP3 inflammasome, which is triggered by mitochondrial dysfunction and oxidative stress [36]. By improving mitochondrial quality and reducing ROS generation, AMPK limits inflammasome activation and downstream cytokine release [13,36]. However, inflammatory signaling in HF is complex and context-dependent, and the relative contribution of AMPK to immune modulation remains incompletely defined.

**Figure 2:**
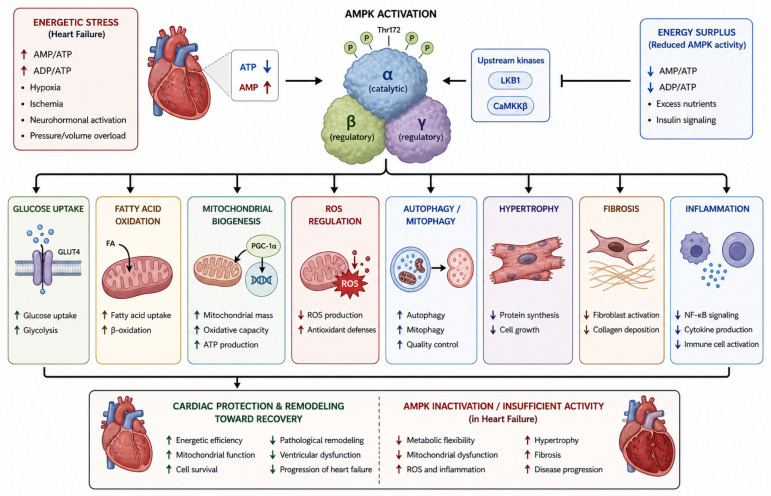
AMPK signaling in heart failure: from energy sensing to cardiac remodelling. Energetic stress in heart failure—including hypoxia, ischemia, neurohormonal activation, and pressure/volume overload—promotes AMPK activation through increases in AMP/ATP and ADP/ATP ratios and upstream kinase signaling via LKB1 and CaMKKβ. Activated AMPK coordinates multiple adaptive metabolic pathways, including enhanced glucose uptake, fatty acid oxidation, mitochondrial biogenesis, autophagy/mitophagy, and antioxidant defenses, while suppressing hypertrophy, fibrosis, inflammation, and pathological remodeling. The figure also highlights the consequences of insufficient AMPK activity, which contributes to mitochondrial dysfunction, oxidative stress, adverse remodeling, and progression of heart failure.

Together, these metabolic and cellular effects position AMPK as a central signaling hub linking energetic stress to cardiac remodeling in HF (Figure 2).

## 4. AMPK in Heart Failure Phenotypes and Diabetes

### 4.1. AMPK Activity in Heart Failure

Regulation of AMPK activity in HF is dynamic and context-dependent rather than uniformly suppressed [37]. In many experimental and clinical settings, AMPK signaling appears insufficient relative to metabolic demand rather than completely inactive, contributing to persistent metabolic imbalance and disease progression [2,3]. The failing heart is characterized by increased glucose uptake, accumulation of metabolic intermediates, mitochondrial dysfunction, and impaired autophagy [2,3]. These changes are associated with oxidative stress, inflammation, fibrosis, and progressive cellular dysfunction. In this context, AMPK may fail to adequately counterbalance nutrient-surplus signaling pathways, particularly those involving mTOR activation [37]. Isoform-specific alterations further complicate this picture. AMPKα2, the predominant cardiac isoform, is generally associated with cardioprotective functions, whereas AMPKα1 is more widely expressed in non-cardiomyocyte cell types [16]. Evidence suggests that AMPKα2 expression may decrease in HF, while AMPKα1 expression increases; however, the functional implications of this shift remain incompletely understood [16]. This raises the possibility that changes in isoform composition, rather than absolute activity alone, influence disease progression.

Importantly, the consequences of altered AMPK signaling are unlikely to be uniform across all forms of HF. Different HF phenotypes are driven by distinct metabolic, inflammatory, and hemodynamic stressors, which may differentially influence AMPK activation, downstream signaling pathways, and therapeutic responsiveness. Emerging evidence suggests that AMPK may function as a central integrator of disease-specific metabolic stress, but the magnitude and biological consequences of its activation vary substantially according to the underlying etiology of HF [9,38]. Overall, AMPK dysfunction in HF appears to reflect an impaired ability to respond appropriately to escalating energetic stress rather than a simple loss of signaling capacity [37].

### 4.2. AMPK in HFrEF and HFpEF

AMPK signaling may play distinct roles across HF phenotypes. In HFpEF, suppression of AMPK-related pathways and activation of nutrient-surplus signaling, including mTOR, are frequently observed [5]. This phenotype is strongly associated with obesity, insulin resistance, and systemic inflammation [4,5]. Adipose tissue–derived factors contribute to oxidative stress, endothelial dysfunction, and fibrosis, linking peripheral metabolic disturbances to myocardial pathology [5]. In contrast, HFrEF is characterized by impaired oxidative metabolism, reduced fatty acid oxidation, and increased reliance on alternative substrates such as glucose and ketone bodies [3]. These alterations contribute to mitochondrial dysfunction and reduced energy availability [3]. While AMPK activation may partially restore metabolic balance, its ability to fully reverse energetic deficits in advanced HFrEF is uncertain [37].

Beyond these two major HF phenotypes, several clinically important HF subtypes exhibit distinct patterns of AMPK dysregulation. In ischemic HF, AMPK is rapidly activated during myocardial ischemia as an adaptive response to ATP depletion, promoting glucose uptake, glycolysis, and cell survival. Experimental studies suggest that AMPK activation may limit infarct size, preserve mitochondrial integrity, and enhance post-ischemic recovery; however, chronic ischemic remodeling may eventually overwhelm these protective mechanisms [39,40]. Thus, in ischemic HF, AMPK primarily functions as an acute stress-response kinase that supports metabolic adaptation during oxygen deprivation.

In obesity-related HF, chronic nutrient excess, adipose tissue inflammation, and lipotoxicity contribute to suppression of AMPK signaling and persistent activation of anabolic pathways such as mTOR [5,38]. Reduced AMPK activity promotes myocardial lipid accumulation, oxidative stress, endothelial dysfunction, and fibrosis, all of which contribute to diastolic dysfunction and HFpEF development. In this setting, restoration of AMPK activity may be particularly important for reversing nutrient-surplus signaling and improving metabolic flexibility [38,41].

Collectively, these observations indicate that AMPK serves distinct pathophysiological roles across HF phenotypes. Whereas restoration of AMPK signaling in HFpEF and obesity-related HF may primarily counteract nutrient overload and inflammation, activation of AMPK in HFrEF and ischemic HF may be more relevant for supporting mitochondrial energetics and adaptation to energy deprivation. These differences suggest that AMPK-targeted strategies may need to be tailored to HF phenotype. In HFpEF, restoring suppressed AMPK signaling and counteracting nutrient-surplus conditions may be beneficial, whereas in HFrEF, improving mitochondrial function and substrate utilization may represent the primary therapeutic objective.

### 4.3. Interaction with Diabetes

Diabetes mellitus is a major contributor to HF development, with diabetic cardiomyopathy representing a distinct pathological entity [6]. Structural and functional alterations, including ventricular remodeling and diastolic dysfunction, often lead initially to HFpEF and may progress to HFrEF over time [42]. In type 2 diabetes, insulin resistance and hyperinsulinemia play central roles in cardiac dysfunction [42]. Suppression of the SIRT1/PGC-1α/AMPK axis, combined with activation of Akt/mTOR signaling, contributes to impaired autophagy, lipid accumulation, and endoplasmic reticulum stress [43]. These processes promote oxidative stress, mitochondrial dysfunction, and inflammation [15,43].

AMPK plays a key role in regulating fatty acid oxidation and maintaining energy balance in diabetic cardiomyocytes [43]. Activation of AMPK has been associated with improved mitochondrial function, reduced myocardial injury, and attenuation of pathological remodeling [15,43]. In addition, AMPK contributes to endothelial function by increasing nitric oxide bioavailability and reducing oxidative stress [15]. Conversely, reduced AMPK activity under hyperglycemic conditions has been linked to impaired insulin signaling and may contribute to disease progression [43]. Diabetic cardiomyopathy deserves particular consideration because AMPK dysregulation appears to be a central pathogenic mechanism rather than merely a secondary consequence of HF. Chronic hyperglycemia, insulin resistance, glucotoxicity, and lipotoxicity collectively suppress AMPK signaling, impair mitochondrial quality control, and promote maladaptive remodeling. Notably, diabetic cardiomyopathy shares several features with obesity-related HF and HFpEF, including systemic inflammation, endothelial dysfunction, and impaired metabolic flexibility, suggesting that AMPK-targeted interventions may have particular relevance in these patient populations [38,42]. Despite its central role, it is unlikely that modulation of AMPK alone is sufficient to fully reverse diabetic cardiomyopathy, given the complexity of underlying metabolic disturbances [6].

The heterogeneous functions of AMPK across HFrEF, HFpEF, ischemic HF, obesity-related HF, and diabetic cardiomyopathy highlight the need for phenotype-specific therapeutic approaches. Future studies should focus on defining disease-specific AMPK signaling networks, identifying biomarkers of AMPK activity, and determining whether selective targeting of AMPK isoforms may improve therapeutic efficacy in distinct HF subtypes.

**Figure 3:**
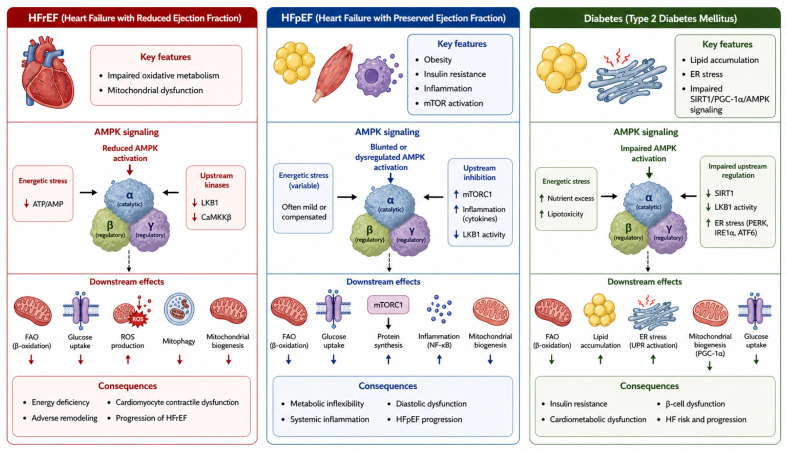
Context-dependent AMPK activity in HFrEF, HFpEF, and diabetes. AMPK signaling differs substantially across major cardiometabolic phenotypes. In heart failure with reduced ejection fraction (HFrEF), reduced AMPK activity is associated with impaired oxidative metabolism and mitochondrial dysfunction. In heart failure with preserved ejection fraction (HFpEF), obesity, insulin resistance, inflammation, and mTOR activation contribute to dysregulated AMPK signaling and metabolic inflexibility. In diabetes, chronic nutrient excess and lipotoxicity impair SIRT1/PGC-1α/AMPK signaling, promoting ER stress, mitochondrial dysfunction, and cardiometabolic injury. The schematic emphasizes that the biological consequences of AMPK activation are highly phenotype-dependent and influenced by upstream metabolic context.

The differential roles of AMPK signaling across HFrEF, HFpEF, and diabetes are summarized in Figure 3.

## 5. Pharmacological Modulation of AMPK

### 5.1. Indirect Activators

#### 5.1.1. Metformin

Metformin is a first-line therapy for type 2 diabetes and has been widely studied for its cardiovascular effects. It indirectly activates AMPK by inhibiting mitochondrial complex I, leading to an increase in the AMP/ATP ratio [29].

In experimental models, metformin enhances glucose uptake, improves mitochondrial efficiency, reduces oxidative stress, and attenuates fibrosis [30,44]. Importantly, many of these effects are not exclusively AMPK-dependent, as metformin also exerts direct mitochondrial and systemic metabolic effects [44].

Clinical evidence suggests that metformin use is associated with lower mortality and reduced HF hospitalization in patients with diabetes [45,46]. However, most data are observational, and residual confounding cannot be excluded. Thus, while metformin may confer cardioprotective effects, the specific contribution of AMPK activation remains uncertain [45].

#### 5.1.2. SGLT2 Inhibitors

SGLT2 inhibitors, including empagliflozin and dapagliflozin, have demonstrated robust clinical benefits in HF across the ejection fraction spectrum [47,48,49]. These agents reduce HF hospitalization and composite cardiovascular outcomes, independent of diabetes status [47,48]. Their primary mechanism involves inhibition of renal glucose reabsorption, but additional effects include improved metabolic efficiency, enhanced ketone utilization, reduced inflammation, and modulation of ion homeostasis [50]. A nutrient deprivation-like state induced by SGLT2 inhibition has been proposed to activate AMPK and SIRT1 signaling pathways [51,52].

Although preclinical studies suggest that AMPK activation contributes to these effects, its role should be considered contributory rather than causal. The clinical benefits of SGLT2 inhibitors are likely multifactorial and cannot be attributed solely to AMPK modulation [50].

### 5.2. Direct Activators

#### 5.2.1. AICAR

AICAR is a commonly used experimental AMPK activator that mimics AMP and promotes AMPK phosphorylation [53]. It enhances fatty acid oxidation and reduces hypertrophic signaling in preclinical models [54]. However, AICAR lacks specificity and exhibits AMPK-independent effects, limiting its translational potential [53]. Its use remains largely restricted to experimental research.

#### 5.2.2. PF-739 and Related Compounds

PF-739 and similar compounds are direct AMPK activators that bind to allosteric regulatory sites [55]. Preclinical studies have demonstrated improved glucose metabolism and enhanced energy utilization [55]. However, these agents have primarily been evaluated in metabolic disease models, and evidence in HF is limited. Their ability to translate metabolic improvements into cardiac benefit remains uncertain.

#### 5.2.3. MK-8722

MK-8722 is a potent pan-AMPK activator that improves systemic glucose homeostasis [56]. However, preclinical studies have reported adverse cardiac effects, including hypertrophy and glycogen accumulation [56]. These findings highlight a key limitation of non-selective AMPK activation and underscore the importance of isoform- and tissue-specific targeting strategies.

### 5.3. Natural Compounds

#### 5.3.1. Resveratrol

Resveratrol is a polyphenolic compound that activates AMPK and exerts cardioprotective effects, including improved endothelial function, reduced oxidative stress, and attenuation of hypertrophic signaling [57]. Clinical studies suggest potential benefits in HF; however, these findings are limited by small sample sizes and short follow-up durations [57]. Low bioavailability further restricts its clinical applicability [58].

#### 5.3.2. Berberine

Berberine has demonstrated AMPK-dependent cardioprotective effects in preclinical models, including improved mitochondrial function, enhanced mitophagy, and reduced apoptosis [59,60]. Despite promising mechanistic data, clinical evidence remains limited, and the contribution of AMPK activation relative to other pathways is not fully defined [61].

A summary of the major pharmacological activators of AMPK is presented in Table 1.

## 6. Non-Pharmacological Modulation of AMPK

### 6.1. Exercise

Exercise is one of the most effective non-pharmacological interventions for the prevention and management of cardiovascular disease and HF. During physical activity, increased ATP consumption elevates intracellular AMP and ADP concentrations, resulting in activation of AMPK through LKB1- and CaMKKβ-dependent mechanisms [62,63]. Acute AMPK activation promotes glucose uptake, fatty acid oxidation, and ATP production, whereas chronic exercise training induces long-term metabolic adaptations including enhanced mitochondrial biogenesis, improved oxidative capacity, and greater metabolic flexibility [63,64].

One of the most important downstream targets of exercise-induced AMPK activation is PGC-1α, a master regulator of mitochondrial biogenesis [65]. Activation of the AMPK–PGC-1α axis improves mitochondrial quantity and quality, which may be particularly beneficial in HF where mitochondrial dysfunction is a hallmark feature [66,67]. Exercise-induced AMPK signaling also promotes antioxidant defense through interactions with NRF2, suppresses inflammatory pathways including NF-κB signaling, and enhances autophagy and mitochondrial quality control [62,63].

Clinical studies suggest that exercise training improves exercise capacity, quality of life, endothelial function, and metabolic health in both HFrEF and HFpEF [68,69,70]. Although AMPK is unlikely to account for all exercise-induced cardiovascular benefits, it is increasingly recognized as a central mediator of these adaptive responses.

### 6.2. Caloric Restriction and Intermittent Fasting

Caloric restriction and intermittent fasting represent powerful physiological activators of AMPK. Reduced nutrient availability increases AMP/ATP ratios and stimulates AMPK signaling, thereby shifting cellular metabolism toward energy conservation and ATP generation [71,72]. Activation of AMPK during fasting suppresses mTOR signaling, enhances autophagy, promotes fatty acid oxidation, and improves mitochondrial quality control [67,71].

These effects may be particularly relevant in HF, where impaired autophagy, mitochondrial dysfunction, and metabolic inflexibility contribute to disease progression. Experimental studies have demonstrated that caloric restriction can attenuate cardiac hypertrophy, reduce oxidative stress, improve insulin sensitivity, and preserve cardiac function [71,72]. Although clinical evidence in HF remains limited, growing interest exists in dietary strategies that mimic fasting-induced AMPK activation.

### 6.3. Weight Loss and Obesity Reduction

Obesity is a major contributor to HFpEF and is frequently associated with insulin resistance, chronic inflammation, and suppression of AMPK signaling [73,74]. Excess nutrient availability promotes activation of anabolic pathways such as mTOR while reducing AMPK activity, thereby contributing to lipid accumulation, oxidative stress, endothelial dysfunction, and myocardial fibrosis.

Weight loss achieved through lifestyle intervention can partially restore AMPK activity, improve insulin sensitivity, reduce systemic inflammation, and enhance metabolic flexibility [73]. Because obesity-related HF and HFpEF are increasingly prevalent, restoration of AMPK signaling through sustained weight reduction may represent an important non-pharmacological therapeutic strategy.

### 6.4. Dietary Interventions and Nutritional Modulation

Dietary composition can influence AMPK activity independently of total caloric intake. Mediterranean dietary patterns, diets rich in polyphenols, and certain ketogenic interventions have been associated with activation of AMPK-related pathways and improvements in metabolic health [75,76].

Several naturally occurring dietary compounds, including resveratrol, quercetin, epigallocatechin gallate (EGCG), and curcumin, have been reported to activate AMPK either directly or indirectly through effects on mitochondrial metabolism and cellular redox state [76,77]. Although many of these compounds are frequently classified as nutraceuticals rather than pharmaceuticals, they illustrate the capacity of dietary factors to influence AMPK-dependent signaling networks.

### 6.5. Circadian Rhythm and Sleep Regulation

Emerging evidence indicates that AMPK interacts closely with circadian clock mechanisms. AMPK regulates components of the molecular clock, including CRY proteins and CLOCK/BMAL1 signaling pathways, thereby linking cellular energy status to circadian regulation [78,79].

Sleep disruption and circadian misalignment have been associated with impaired AMPK signaling, insulin resistance, metabolic dysfunction, and increased cardiovascular risk [80]. Given the high prevalence of sleep disorders among patients with HF, circadian regulation may represent an underappreciated mechanism through which AMPK influences cardiovascular health.

### 6.6. Emerging Environmental Stressors

Additional non-pharmacological stimuli capable of activating AMPK include cold exposure, heat therapy, and hypoxic conditioning [80,81]. These interventions increase energetic demand and induce adaptive stress responses that may stimulate AMPK signaling. Although evidence supporting their role in HF management remains limited, they further illustrate the broad responsiveness of AMPK to physiological stress.

Collectively, these observations position AMPK as a central mediator through which lifestyle interventions exert cardiovascular benefits. Exercise remains the most extensively studied and clinically validated non-pharmacological activator of AMPK; however, caloric restriction, weight loss, dietary modification, circadian regulation, and environmental conditioning may also contribute to improved metabolic homeostasis, mitochondrial function, and cardiovascular resilience. Future studies should determine whether combining lifestyle-based strategies with pharmacological AMPK activation may provide additive or synergistic benefits in HF management.

## 7. Crosstalk with Cardioprotective Pathways

### 7.1. AMPK and mTOR Signaling

The mechanistic target of rapamycin (mTOR) pathway is a central regulator of cellular growth and metabolism, integrating signals related to nutrient availability, growth factors, and energy status [82,83]. mTOR exists in two complexes: mTORC1, which promotes protein synthesis and inhibits autophagy, and mTORC2, which regulates cell survival and cytoskeletal organization [82,83].

AMPK interacts with this pathway primarily through inhibition of mTORC1. Under conditions of energetic stress, AMPK phosphorylates regulatory proteins such as tuberous sclerosis complex 2 (TSC2) and Raptor, leading to suppression of mTORC1 activity [19]. This shift reduces anabolic processes and promotes autophagy, facilitating cellular adaptation [19]. The AMPK–mTOR axis, therefore, functions as a metabolic switch that determines whether cardiomyocytes prioritize growth or repair. While inhibition of mTORC1 may be beneficial in limiting pathological hypertrophy, excessive or prolonged suppression may impair adaptive growth and cellular recovery [32]. Therapeutic strategies should therefore aim to restore balance rather than achieve complete inhibition.

### 7.2. AMPK and cGMP-PKG Signaling

The nitric oxide (NO)-cGMP-protein kinase G (PKG) pathway plays a critical role in cardiovascular protection, regulating vascular tone, myocardial stiffness, and cellular signaling [84,85]. Activation of endothelial nitric oxide synthase (eNOS) increases NO production, leading to stimulation of soluble guanylyl cyclase and subsequent cGMP generation [85].

AMPK interacts with this pathway primarily through modulation of eNOS activity. Activation of AMPK enhances phosphorylation of eNOS, increasing NO bioavailability and supporting downstream PKG signaling [15]. This interaction contributes to improved endothelial function, reduced myocardial stiffness, and attenuation of hypertrophic signaling [84].

Compared with other AMPK-associated pathways, this interaction is less direct and reflects the integration of metabolic and vascular signaling. It is particularly relevant in HFpEF, where impaired NO-cGMP-PKG signaling contributes to diastolic dysfunction [84].

### 7.3. AMPK and SIRT1/NAD^+^ Metabolism

AMPK is closely linked to nicotinamide adenine dinucleotide (NAD^+^)-dependent signaling through interaction with sirtuins, particularly SIRT1 [86]. This relationship forms a reciprocal regulatory loop that integrates metabolic status with transcriptional control [86]. AMPK promotes NAD^+^ biosynthesis by upregulating nicotinamide phosphoribosyltransferase (NAMPT), thereby enhancing SIRT1 activity [87]. In turn, SIRT1 activates AMPK through deacetylation of upstream kinases such as LKB1 [86]. This feedback loop supports mitochondrial biogenesis, oxidative metabolism, and resistance to cellular stress through downstream mediators including PGC-1α [86]. This pathway is particularly relevant in aging, diabetes, and HFpEF, where NAD^+^ depletion and impaired nutrient sensing contribute to disease progression [4,86]. However, the clinical impact of targeting NAD^+^ metabolism in HF remains uncertain. However, it should be mentioned that NAD+ regulates the number of cardiac ATP-sensitive potassium (KATP) channels, conferring cardioprotection [88,89,90], which is known to be beneficial in heart failure [91].

### 7.4. AMPK and ATP-Sensitive K^+^ (KATP) Channels

AMPK is a key metabolic sensor in cardiomyocytes that links cellular energy status to the regulation of ATP-sensitive potassium (KATP) channels, which are composed of the pore-forming Kir6.2 subunits and the regulatory SUR2A subunit [92,93]. During metabolic stress, such as ischemia or increased workload, rising AMP/ATP ratios activate AMPK, which in turn enhances KATP channel activity through direct effects on channel gating and by promoting channel trafficking to the sarcolemma [92].

In addition, AMPK has been shown to upregulate SUR2A expression, increasing the number of functional KATP channels [94]. The opening of these channels shortens the cardiac action potential, reduces calcium influx, and lowers ATP consumption, thereby protecting cardiomyocytes from ischemic and metabolic injury [93]. By preserving cellular energy balance, limiting calcium overload, and enhancing resistance to stress, AMPK-mediated activation of KATP channels may help maintain cardiac function during metabolic stress [37,92].

### 7.5. AMPK and Mitochondrial Signaling

Mitochondria serve as both targets and regulators of AMPK signaling. Changes in mitochondrial function directly influence AMPK activation through alterations in cellular energy charge, reflected by AMP/ATP and ADP/ATP ratios [3,15].

In addition to energy-dependent activation, mitochondrial stress can stimulate AMPK through calcium-dependent pathways involving CaMKKβ, as well as through redox-sensitive mechanisms associated with reactive oxygen species [13,15]. Once activated, AMPK promotes mitochondrial quality control by enhancing biogenesis, regulating mitophagy, and maintaining efficient turnover of damaged organelles [15,31]. These processes are essential for preserving bioenergetic capacity under stress conditions. However, excessive activation of mitochondrial clearance pathways may become maladaptive if not balanced by adequate biogenesis [13,31].

## 8. Clinical and Translational Evidence

### 8.1. Metformin and Heart Failure Outcomes

Metformin is widely used in the management of type 2 diabetes and exerts part of its metabolic effect through indirect activation of AMPK [95]. Mechanistically, it inhibits mitochondrial complex I, leading to an increase in the AMP/ATP ratio and subsequent activation of AMPK-dependent pathways [96].

Observational studies have consistently reported associations between metformin use and reduced mortality and heart failure hospitalization in patients with diabetes [97,98,99]. Some evidence also suggests improvements in myocardial structure and function, including reduced left ventricular mass and improved diastolic parameters [100].

Although these findings are encouraging, the clinical evidence supporting metformin specifically as an AMPK-targeted therapy for HF remains indirect. Most studies have been observational, and relatively few randomized trials have evaluated HF-specific outcomes. Furthermore, metformin exerts numerous AMPK-independent effects, including alterations in mitochondrial metabolism, gut microbiota composition, and inflammatory signaling pathways [95,96]. Consequently, it remains difficult to determine the extent to which AMPK activation directly contributes to the observed cardiovascular benefits. An additional unresolved question is whether metformin provides similar benefits across different HF phenotypes, particularly HFpEF, where metabolic dysfunction may play a more prominent pathogenic role.

### 8.2. SGLT2 Inhibitors and Indirect AMPK Activation

SGLT2 inhibitors have emerged as a cornerstone therapy for HF across the ejection fraction spectrum. Large randomized trials have demonstrated consistent reductions in heart failure hospitalization and composite cardiovascular outcomes [47,51].

The DAPA-HF trial demonstrated that dapagliflozin significantly reduced the composite risk of worsening HF or cardiovascular death in patients with HFrEF regardless of diabetes status [47]. Similarly, the EMPEROR-Reduced trial showed that empagliflozin reduced the risk of cardiovascular death or HF hospitalization in patients with HFrEF and slowed the decline in renal function [51]. Importantly, the benefits of SGLT2 inhibition extended beyond HFrEF. In EMPEROR-Preserved, empagliflozin became the first therapy to demonstrate a significant reduction in the composite endpoint of cardiovascular death or HF hospitalization in patients with HFpEF [48]. These findings were subsequently supported by the DELIVER trial, which confirmed the efficacy of dapagliflozin across a broad spectrum of left ventricular ejection fractions [49].

Mechanistically, these agents induce glucosuria and reduce insulin levels, creating a metabolic state resembling nutrient deprivation [101]. This shift has been proposed to activate AMPK and SIRT1 signaling pathways, alongside improvements in mitochondrial function, inflammation, and substrate utilization [51,102].

Nevertheless, direct evidence linking SGLT2 inhibitor efficacy to AMPK activation in humans remains limited. Multiple complementary mechanisms have been proposed, including osmotic diuresis, improved cardiac energetics, reduced inflammation, modulation of ion homeostasis, enhanced ketone body utilization, and renal protection [51,101]. AMPK activation should therefore be viewed as one component of a broader cardioprotective network rather than the sole mediator of clinical benefit. Clarifying the relative contribution of AMPK signaling to SGLT2 inhibitor efficacy remains an important translational challenge.

### 8.3. Translational Gaps and Unresolved Questions

Despite strong mechanistic evidence supporting AMPK as a therapeutic target, direct clinical activation remains limited [103]. Currently available therapies modulate AMPK indirectly, and their benefits cannot be specifically attributed to AMPK activation.

A major translational challenge is the absence of highly selective AMPK-targeted therapies that have demonstrated efficacy in large-scale cardiovascular outcome trials. Although numerous AMPK activators have shown promising results in experimental models, relatively few have advanced to late-stage clinical development. Consequently, the translational pathway from mechanistic discovery to therapeutic implementation remains incomplete.

A major challenge lies in the complexity of AMPK signaling, including isoform diversity and tissue-specific effects [104]. Non-selective activation may produce divergent outcomes, including adverse cardiac remodeling or metabolic disturbances [18].

Genetic and pharmacological evidence further highlights these concerns. Preclinical studies of pan-AMPK activators, such as MK-8722, have reported adverse cardiac effects including hypertrophy and glycogen accumulation [56].

Several additional questions remain unresolved. First, it is unclear which HF phenotypes are most likely to benefit from therapeutic AMPK modulation. Although AMPK activation may be particularly relevant in obesity-related HF, diabetic cardiomyopathy, and HFpEF, direct comparative clinical evidence is lacking. Second, biomarkers capable of quantifying AMPK activation in patients are not well established, limiting the ability to assess target engagement and personalize therapy. Third, the optimal timing, intensity, and duration of AMPK activation remain uncertain. As discussed previously, excessive or prolonged activation may produce maladaptive effects, suggesting that therapeutic benefit may depend on maintaining AMPK activity within a specific physiological range.

Finally, future clinical development should move beyond demonstrating associations between AMPK activation and cardiovascular benefit toward establishing causal relationships through biomarker-guided studies, isoform-selective approaches, and carefully designed mechanistic clinical trials. Addressing these challenges will be essential for determining whether AMPK can ultimately become a direct therapeutic target in HF rather than simply a mediator of the effects of existing therapies.

## 9. Challenges in Targeting AMPK

### 9.1. Isoform Specificity

AMPK exists as multiple heterotrimeric complexes with distinct isoform compositions [103]. In the heart, AMPKα2 predominates in cardiomyocytes and is closely associated with metabolic adaptation and cardioprotection, whereas AMPKα1 is more widely expressed in non-cardiomyocyte cell populations and peripheral tissues [105].

Although AMPK activation is generally considered beneficial, emerging evidence indicates that its biological effects are highly context-dependent and may vary according to isoform composition, cell type, disease stage, and duration of activation. Non-selective activation of AMPK may therefore produce competing or even opposing effects across different tissues. While activation of AMPKα2 may enhance myocardial resilience and improve energetic homeostasis, activation of AMPKα1 in fibroblasts, immune cells, or extracardiac tissues may influence inflammation, fibrosis, and systemic metabolism in ways that are not always beneficial [105]. Furthermore, genetic studies suggest that different AMPK isoforms may regulate distinct signaling pathways, raising concerns that indiscriminate activation of all AMPK complexes could lead to unintended consequences [16]. These observations highlight the need for a greater understanding of isoform-specific AMPK biology before highly targeted therapeutic strategies can be developed.

### 9.2. Acute Versus Chronic Activation

The effects of AMPK activation depend strongly on temporal dynamics. Acute activation during energetic stress promotes adaptive responses, including improved substrate utilization, enhanced mitochondrial function, and activation of cellular survival pathways [103].

In contrast, chronic or sustained AMPK activation may not always be beneficial. Although prolonged activation can maintain metabolic homeostasis under certain conditions, excessive suppression of anabolic signaling pathways may impair normal cellular growth, protein synthesis, and tissue repair [106,107]. Experimental studies have demonstrated that persistent AMPK activation may contribute to excessive autophagy, reduced protein translation through inhibition of mTOR signaling, and altered myocardial remodeling [107,108]. Moreover, some evidence suggests that chronic AMPK activation may become maladaptive in advanced HF when severe energetic deficits cannot be corrected solely through metabolic reprogramming. In such settings, continued activation of catabolic pathways could potentially exacerbate myocardial dysfunction rather than restore homeostasis [107].

Another unresolved issue concerns the temporal relationship between AMPK activation and disease progression. It remains unclear whether AMPK activation exerts equivalent benefits during early-stage disease, advanced HF, ischemic injury, and metabolic cardiomyopathy. Consequently, the timing of therapeutic intervention may be as important as the degree of AMPK activation itself.

### 9.3. Risk of Excessive Catabolism

AMPK promotes catabolic pathways while suppressing anabolic processes [103]. While this shift is beneficial in acute stress conditions, prolonged activation may result in excessive protein degradation and cellular atrophy [9].

Increasing evidence indicates that excessive or prolonged AMPK activation may promote maladaptive catabolic responses in certain pathological settings. Sustained inhibition of mTOR signaling may impair protein synthesis and regenerative processes required for maintenance of myocardial structure and function [108]. Likewise, excessive stimulation of autophagy could potentially lead to autophagic cell death or depletion of essential cellular components if not appropriately regulated [26]. Although these phenomena remain incompletely understood in human HF, they illustrate the importance of maintaining a balance between metabolic adaptation and structural preservation.

Importantly, several observations challenge the notion that AMPK activation is universally protective. For example, mutations in genes encoding AMPK subunits have been linked to inherited cardiomyopathies characterized by glycogen accumulation, ventricular hypertrophy, and conduction abnormalities [109]. These findings suggest that dysregulated AMPK signaling can contribute directly to cardiac pathology under specific circumstances. Furthermore, some pharmacological AMPK activators exert substantial off-target effects, making it difficult to distinguish AMPK-dependent benefits from unrelated biological actions [110].

An important example illustrating the potential detrimental consequences of dysregulated AMPK signaling is PRKAG2 cardiomyopathy, a rare inherited cardiac disorder caused by mutations in the gene encoding the AMPK γ2 regulatory subunit. Unlike the reduced AMPK activity frequently observed in metabolic disease and HF, PRKAG2 mutations often result in constitutive or aberrant AMPK activation, leading to excessive glycogen accumulation within cardiomyocytes, ventricular hypertrophy, conduction abnormalities, and an increased risk of arrhythmias. These clinical observations demonstrate that AMPK activation is not universally beneficial and that both insufficient and excessive signaling may be pathological [111,112]. The PRKAG2 syndrome therefore provides a compelling human model highlighting the importance of tightly regulated AMPK activity and supports the concept that therapeutic strategies should aim to restore physiological AMPK signaling rather than indiscriminately maximize its activation.

Collectively, these findings emphasize that AMPK should not be viewed as an inherently beneficial therapeutic target under all conditions. Rather, its effects appear to be highly dependent on disease context, cell type, activation magnitude, isoform specificity, and treatment duration. Future studies should focus on defining the therapeutic window for AMPK modulation, identifying biomarkers of appropriate target engagement, and determining which patient populations are most likely to benefit from AMPK-targeted interventions.

## 10. Future Directions

### 10.1. Isoform-Selective Modulation

Future therapeutic strategies are likely to focus on selective activation of AMPK complexes containing the α2 isoform, which is predominant in cardiomyocytes [104]. Advances in structural biology and drug design may enable the development of compounds that target specific regulatory interfaces and improve isoform selectivity [103].

### 10.2. Tissue-Specific Targeting

Emerging approaches aim to restrict AMPK modulation to the myocardium. These include nanoparticle-based delivery systems, targeted drug delivery platforms, and prodrugs activated under pathological conditions. In addition, modulation of tissue-specific upstream regulators such as LKB1 and CaMKKβ represents a promising strategy to enhance selectivity. Such approaches may improve therapeutic efficacy while minimizing systemic effects and off-target metabolic consequences [113,114,115].

### 10.3. Combination Therapies

Given the multifactorial nature of HF, combination therapies targeting complementary pathways may provide greater benefit than single-agent approaches [40]. Co-targeting AMPK and pathways such as cGMP-PKG or combining AMPK modulation with SGLT2 inhibitors may offer synergistic effects by addressing both metabolic and hemodynamic components of disease [74,101].

### 10.4. Precision Medicine Approaches

**Figure 4:**
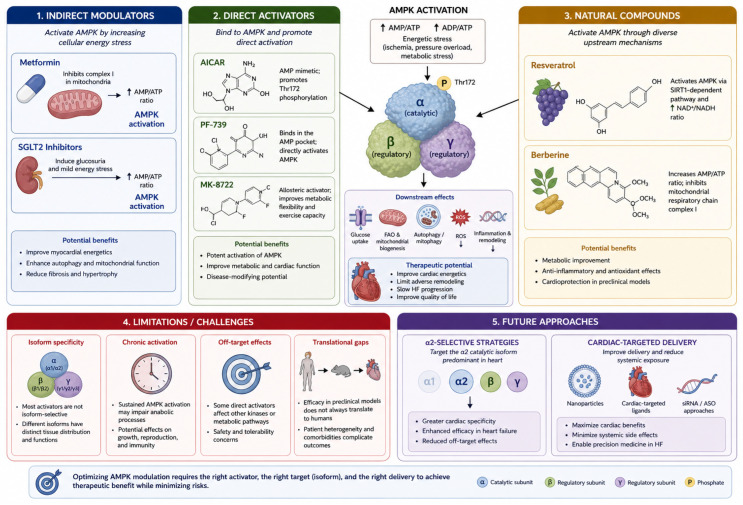
Therapeutic modulation of AMPK: opportunities and challenges. Multiple pharmacological and nutraceutical approaches modulate AMPK activity through distinct mechanisms. Indirect modulators such as metformin and SGLT2 inhibitors activate AMPK secondary to alterations in cellular energy balance, whereas direct activators including AICAR, PF-739, and MK-8722 bind AMPK and promote catalytic activation. Natural compounds such as resveratrol and berberine stimulate AMPK through upstream metabolic and redox pathways. The figure also summarizes key limitations of AMPK-targeted therapy, including limited isoform specificity, risks associated with chronic activation, off-target effects, and translational challenges. Future therapeutic strategies are expected to focus on α2-selective and cardiac-targeted AMPK modulation to maximize efficacy while minimizing systemic toxicity.

HF is a heterogeneous syndrome, and uniform therapeutic strategies are unlikely to be effective across all patients. Integration of multi-omics profiling, biomarker development, and advanced imaging may enable identification of patient subgroups with dysregulated metabolic pathways who may benefit most from AMPK-targeted therapies. This approach supports a shift toward individualized treatment strategies based on underlying disease mechanisms and metabolic phenotyping [8,116,117]. Current pharmacological approaches and future precision-targeting strategies for AMPK modulation are summarized in Figure 4.

## 11. Conclusions

Heart failure is increasingly recognized as a disorder of impaired metabolic flexibility and disrupted energy homeostasis. AMPK plays a central role in integrating energy sensing with adaptive cellular responses, influencing metabolism, mitochondrial function, autophagy, and survival pathways.

While experimental evidence supports its cardioprotective potential, AMPK signaling is highly context-dependent. Its effects vary according to isoform composition, disease stage, and tissue specificity. Importantly, indirect modulation through established therapies such as SGLT2 inhibitors and metformin highlights the clinical relevance of metabolic targeting but does not establish AMPK as the sole mediator of therapeutic benefit.

Direct pharmacological activation remains challenging due to risks associated with non-selective or sustained activation, including excessive catabolism and adverse remodeling. Future strategies will require precise, context-dependent modulation that reflects the complexity of AMPK biology.

Advances in drug design, targeted delivery systems, and precision medicine approaches may enable more effective exploitation of AMPK signaling in HF. Ultimately, the therapeutic value of AMPK will depend on the ability to align its modulation with the specific metabolic and molecular characteristics of individual patients.

## Figures and Tables

**Table 1:** Pharmacological modulators of AMPK and their translational potential in heart failure.

Agent/Class	AMPK Modulation	Primary Mechanism	Evidence for CV/HF Benefit	Major Limitations	Translational Status
Metformin	Indirect activator	Inhibits mitochondrial complex I; increases AMP/ATP ratio	Reduced mortality/HF hospitalization in observational studies; favorable metabolic effects [29,44,45,46]	Observational evidence; AMPK-independent effects	Established therapy for T2DM
SGLT2 inhibitors	Indirect activator	Nutrient deprivation-like state; AMPK/SIRT1 activation	reduced HF events [47,48,49,51]	Benefits are multifactorial; AMPK contribution is uncertain	Established HF therapy
AICAR	Direct activator	AMP mimetic promoting AMPK phosphorylation	Improves fatty acid oxidation and reduces hypertrophy in preclinical models [53,54]	Poor specificity; AMPK-independent actions	Experimental
PF-739 and related compounds	Direct activator	Allosteric activation of AMPK	Improves glucose metabolism in preclinical studies [55]	Limited HF-specific evidence	Preclinical
MK-8722	Pan-AMPK activator	Potent systemic AMPK activation	Improves glucose homeostasis [56]	Cardiac hypertrophy and glycogen accumulation	Development limited by safety concerns
Resveratrol	Natural activator	AMPK/SIRT1 activation; antioxidant effects	Improves endothelial function and reduces oxidative stress [57,58]	Low bioavailability; limited clinical evidence	Nutraceutical
Berberine	Natural activator	AMPK activation; enhanced mitophagy and mitochondrial function	Cardioprotective effects in preclinical models [59,60,61]	Limited clinical evidence	Early translational stage
Emerging isoform-selective activators	Direct activator	Selective targeting of AMPK isoforms	Strong mechanistic rationale [16]	Lack of clinical outcome data	Experimental

## Data Availability

No new data were created or analyzed. Data sharing does not apply to the article.

## Abbreviations

The following abbreviations are used in this manuscript:

ADP	Adenosine Diphosphate
AICAR	5-Aminoimidazole-4-Carboxamide Ribonucleotide
Akt	Protein Kinase B
AMP	Adenosine Monophosphate
AMPK	AMP-Activated Protein Kinase
AMPKα	AMPK Alpha Catalytic Subunit
AMPKα1	AMPK Alpha-1 Isoform
AMPKα2	AMPK Alpha-2 Isoform
ATP	Adenosine Triphosphate
BMAL1	Brain and Muscle ARNT-Like Protein 1
CaMKKβ	Ca^2+^/Calmodulin-Dependent Protein Kinase Kinase Beta
cGMP	Cyclic Guanosine Monophosphate
CLOCK	Circadian Locomotor Output Cycles Kaput
CRY	Cryptochrome
DAPA-HF	Dapagliflozin and Prevention of Adverse Outcomes in Heart Failure
EGCG	Epigallocatechin Gallate
ER	Endoplasmic Reticulum
eNOS	Endothelial Nitric Oxide Synthase
GLUT1	Glucose Transporter 1
GLUT4	Glucose Transporter 4
GSK3β	Glycogen Synthase Kinase 3 Beta
HF	Heart Failure
HFmrEF	Heart Failure with Mildly Reduced Ejection Fraction
HFpEF	Heart Failure with Preserved Ejection Fraction
HFrEF	Heart Failure with Reduced Ejection Fraction
HO-1	Heme Oxygenase-1
KATP	ATP-Sensitive Potassium Channel
Kir6.2	Inward Rectifier Potassium Channel 6.2
LKB1	Liver Kinase B1
miR-29	MicroRNA-29
mTOR	Mechanistic Target of Rapamycin
mTORC1	Mechanistic Target of Rapamycin Complex 1
mTORC2	Mechanistic Target of Rapamycin Complex 2
NAD^+^	Nicotinamide Adenine Dinucleotide
NAMPT	Nicotinamide Phosphoribosyltransferase
NF-κB	Nuclear Factor Kappa B
NLRP3	NLR Family Pyrin Domain Containing 3
NO	Nitric Oxide
NRF2	Nuclear Factor Erythroid 2–Related Factor 2
PF-739	Direct AMPK Activator Compound
PGC-1α	Peroxisome Proliferator-Activated Receptor Gamma Coactivator-1 Alpha
PINK1	PTEN-Induced Kinase 1
PKG	Protein Kinase G
Raptor	Regulatory-Associated Protein of mTOR
ROS	Reactive Oxygen Species
SGLT2	Sodium-Glucose Cotransporter 2
SIRT1	Sirtuin 1
SUR2A	Sulfonylurea Receptor 2A
T2DM	Type 2 Diabetes Mellitus
TAK1	Transforming Growth Factor-β-Activated Kinase 1
TGF-β	Transforming Growth Factor Beta
Thr172	Threonine 172
TSC2	Tuberous Sclerosis Complex 2
UKPDS	United Kingdom Prospective Diabetes Study
ULK1	Unc-51-Like Kinase 1
